# Structural and Functional Maturation of Rat Primary Motor Cortex Layer V Neurons

**DOI:** 10.3390/ijms21176101

**Published:** 2020-08-24

**Authors:** Bruno Benedetti, Dominik Dannehl, Jan Maximilian Janssen, Corinna Corcelli, Sébastien Couillard-Després, Maren Engelhardt

**Affiliations:** 1 Spinal Cord Injury and Tissue Regeneration Center Salzburg (SCI-TReCS), 5020 Salzburg, Austria; dominik.dannehl@medma.uni-heidelberg.de (D.D.); s.couillard-despres@pmu.ac.at (S.C.-D.); 2 Institute of Experimental Neuroregeneration, Paracelsus Medical University, 5020 Salzburg, Austria; 3 Austrian Cluster for Tissue Regeneration, 1000 Vienna, Austria; 4 Institute of Neuroanatomy, Medical Faculty Mannheim, Heidelberg University, 68167 Mannheim, Germany; Maximilian.Janssen@medma.uni-heidelberg.de (J.M.J.); corinna.corcelli@medma.uni-heidelberg.de (C.C.); 5 Mannheim Center for Translational Neuroscience (MCTN), Medical Faculty Mannheim, Heidelberg University, 68167 Mannheim, Germany

**Keywords:** axon initial segment (AIS), maturation, motor cortex, development, patch clamp, motor neurons

## Abstract

Rodent neocortical neurons undergo prominent postnatal development and maturation. The process is associated with structural and functional maturation of the axon initial segment (AIS), the site of action potential initiation. In this regard, cell size and optimal AIS length are interconnected. In sensory cortices, developmental onset of sensory input and consequent changes in network activity cause phasic AIS plasticity that can also control functional output. In non-sensory cortices, network input driving phasic events should be less prominent. We, therefore, explored the relationship between postnatal functional maturation and AIS maturation in principal neurons of the primary motor cortex layer V (M1LV), a non-sensory area of the rat brain. We hypothesized that a rather continuous process of AIS maturation and elongation would reflect cell growth, accompanied by progressive refinement of functional output properties. We found that, in the first two postnatal weeks, cell growth prompted substantial decline of neuronal input resistance, such that older neurons needed larger input current to reach rheobase and fire action potentials. In the same period, we observed the most prominent AIS elongation and significant maturation of functional output properties. Alternating phases of AIS plasticity did not occur, and changes in functional output properties were largely justified by AIS elongation. From the third postnatal week up to five months of age, cell growth, AIS elongation, and functional output maturation were marginal. Thus, AIS maturation in M1LV is a continuous process that attunes the functional output of pyramidal neurons and associates with early postnatal development to counterbalance increasing electrical leakage due to cell growth.

## 1. Introduction

The axon initial segment (AIS) is an electrogenic microdomain located at the proximal axon. It is endowed with a high density of voltage-gated ion channels and responsible for the initiation of action potentials (AP) [1,2,3]. AIS morphology and molecular composition can dynamically adapt to control neuronal intrinsic excitability and efficiency of input–output conversion, i.e., how likely neurons are to fire APs in response to depolarization [4,5,6,7].

Changes in network activity can prompt AIS plasticity [8,9,10,11]. In simplified terms, longer AISs contribute to increased intrinsic neuronal excitability, while shorter AISs correspond to decreased excitability [8]. Furthermore, changes in cell volume and volume-related properties affect AIS morphology and location [12]. Thus, short AISs are better suited for input–output conversion in small neurons, whereas long AISs are likely better suited for large neurons [12,13].

During corticogenesis, initially small and rather spherical neuronal precursors become large and ramified neurons. At the same time, growing neurons integrate increasing amounts of synaptic input. In rodents, both processes are prominent after birth [14]. Thus, early postnatal development of cortical neurons involves discrete synchronous intrinsic and extrinsic factors, chiefly the increase of cellular volume and synaptic input, which should affect AIS maturation. On one hand, progressively increasing cell volume and branching should promote gradual homeostatic AIS elongation. On the other hand, extrinsic factors such as developing local network activity and the emergence of cortical inhibition should trigger AIS plasticity and add inhomogeneity to the process of AIS maturation.

In the case of neurons maturing in the sensory cortex, postnatal AIS plasticity follows a multi-phasic process shaped by the onset of sensory input, and it is, therefore, susceptible to sensory deprivation [8,15,16,17,18]. In contrast, the more linear and monophasic process of postnatal AIS elongation described in some non-sensory areas may reflect the subordinated influence of external input to these brain regions [17]. Therefore, we hypothesized that maturing output neurons of the primary motor cortex (M1) undergo a monophasic process of AIS elongation during early postnatal development, which accompanies the progressive increase in cell volume and leads to gradual maturation of physiological output. However, we could not exclude that developmental AIS plasticity during M1 postnatal maturation will also be influenced by the growing sensory input [19]. In either circumstance, once mature neuronal morphology and connectivity are attained, AISs of M1 neurons should reach their final length for efficient output generation in the adult brain.

In this study, we addressed postnatal AIS development and intrinsic physiological properties of rat M1 layer V pyramidal neurons (M1LV neurons), which are the main recipients of columnar inter-laminar excitatory synaptic input and a source of corticofugal output [20]. Based on the course of rat postnatal development [14] and on the dynamics of synaptic remodeling [21], our analysis focused more extensively on the development of AISs and the functional output of M1LV in the early postnatal weeks. After this critical period, progressive refinement of connectivity and further intrinsic functional maturation are expected to endure until approximately two months of age [22].

## 2. Results

### 2.1. Increase in Cell Size and AIS Elongation Are Synchronized in the First Postnatal Weeks

**Figure 1:**
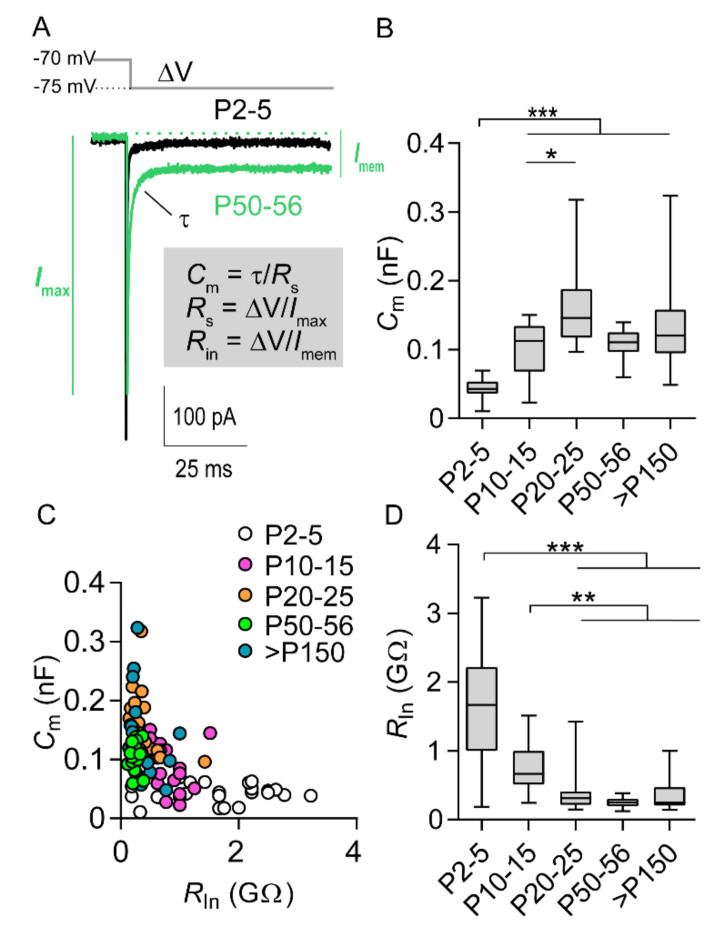
Development of intrinsic membrane properties in M1 layer V (M1LV) pyramidal neurons. (**A**) Representative current elicited by a −5 mV hyperpolarizing voltage step applied at −70 mV. Black and green traces show typical amplitudes of maximal current (*I*_max_) and of steady-state membrane leak current (*I*_mem_) for P2–5 (black) and P50–56 (green) neurons. Cell capacitance (*C*_m_) was calculated as ratio between time-constant (τ) and series resistance (*R*_s_ = 5 mV/*I*_max_). *R_In_* was calculated as the ratio between step voltage (−5 mV) and *I*_mem_. (**B**) *C*_m_ significantly increased with age. (**C**) *C*_m_ and *R_In_* were inversely related. (**D**) *R_In_* significantly decreased with age. * *p* < 0.05; ** *p* < 0.01; *** *p* < 0.001; P2–5: *n* = 24; P10–15: *n* = 21; P20–25: *n* = 21; P50–56: *n* = 19; P > 150: *n* = 20 (more details in Table S1, Supplementary Materials).

The neocortex of rodents undergoes prominent structural and functional maturation after birth [14]. Voltage clamp experiments carried out on M1LV neurons in rat acute brain slices revealed an age-dependent increase in cell capacitance (*C*_m_), which is an electrical correlate to cell size, thus confirming that maturing neurons increased in size in the early postnatal period. Significant differences were detected when comparing the *C*_m_ of neurons of the first postnatal week to neurons of any older age group. Moreover, significant differences were detected comparing P10–15 neurons to P20–25 neurons. However, no significant differences were observed between age groups older than three weeks (Figure 1A,B; Table S1, Supplementary Materials). During the same period, the input resistance (*R*_In_) was inversely related to the increasing *C*_m_ (Figure 1C), implying that the larger neurons were electrically leakier. Accordingly, *R_In_* decreased with age (Figure 1A,C; Table S1, Supplementary Materials), and significant differences were detected comparing neurons of the first two postnatal weeks to neurons older than three weeks. In contrast, no significant differences were detected between age groups older than three weeks or between neurons of the first and second postnatal week (Figure 1A,C,D; Table S1, Supplementary Materials). In line with the period of most prominent growth, M1LV neurons also underwent hyperpolarization of the resting membrane potential (*E*_rest_). Age-related hyperpolarization (approximately 12 mV, Table S1, Supplementary Materials) was only significant when comparing the youngest group to older age groups. In contrast, no significant differences in *E*_rest_ were detected between age groups older than one week.

**Figure 2:**
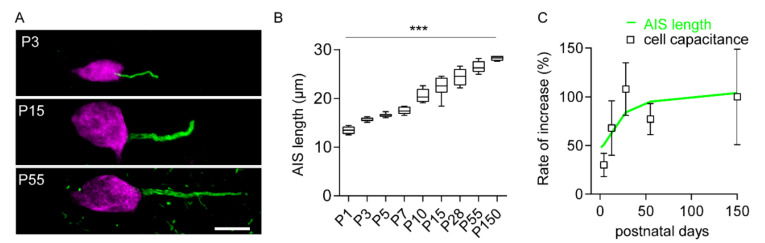
Developmental axon initial segment (AIS) elongation in M1LV pyramidal neurons. (**A**) Immunofluorescent labeling highlighting age-dependent AIS elongation from P3 to P15 to P55. AIS indicated by βIV-spectrin (green) and neuronal somata by NeuN (magenta) in M1LV. Puncta in the background at P55 indicate the emergence of nodes of Ranvier. For clarity, cells were isolated from their respective background, while all other cells were eliminated and channels were optimized for contrast and brightness (Adobe Photoshop). Scale bar = 10 µm. (**B**) Quantification of AIS length (*n* = 6 rats/age group, *n* = 600 AIS/age group); *** *p* < 0.001. Note: in this graph, for clarity of representation, *p* describes one-way Anova. *p*-Values of the post hoc test are detailed in Table S3 (Supplementary Materials). (**C**) The most prominent AIS elongation (green line) occurred within the third postnatal week, in line with the largest increase in *C*_m_ (represented as average ± SD, see also Table S1, Supplementary Materials).

AIS measurements based on βIV-spectrin immunodetection (Figure 2A) allowed testing whether the growth of M1LV neurons was associated with proportional AIS elongation. Measurements revealed that the AISs of maturing neurons elongated with age, from the first postnatal day (length at P1; ~13 µm) to the fifth month (length at P > 150; ~28 µm; Figure 2A,B; Tables S2 and S3, Supplementary Materials). Notably, the most prominent AIS elongation coincided with the most prominent postnatal increase in *C*_m_, as more than 80% of the total AIS elongation (normalized to P150) happened in the first three postnatal weeks (Figure 2C). AIS elongation occurred along with AIS widening and with a small shift toward the soma (Table S2, Supplementary Materials).

### 2.2. Postnatal Development of Functional Output Mirrors Prominent AIS Elongation

In light of these findings and the relationship between AIS length and intrinsic cell excitability, crucial steps of functional output development in M1LV neurons are expected before the third postnatal week. Accordingly, the functional output of M1LV neurons was analyzed with current clamp experiments in five age groups (from P0 to >P150) to closely monitor the changes of intrinsic excitability and AP firing after birth.

**Figure 3:**
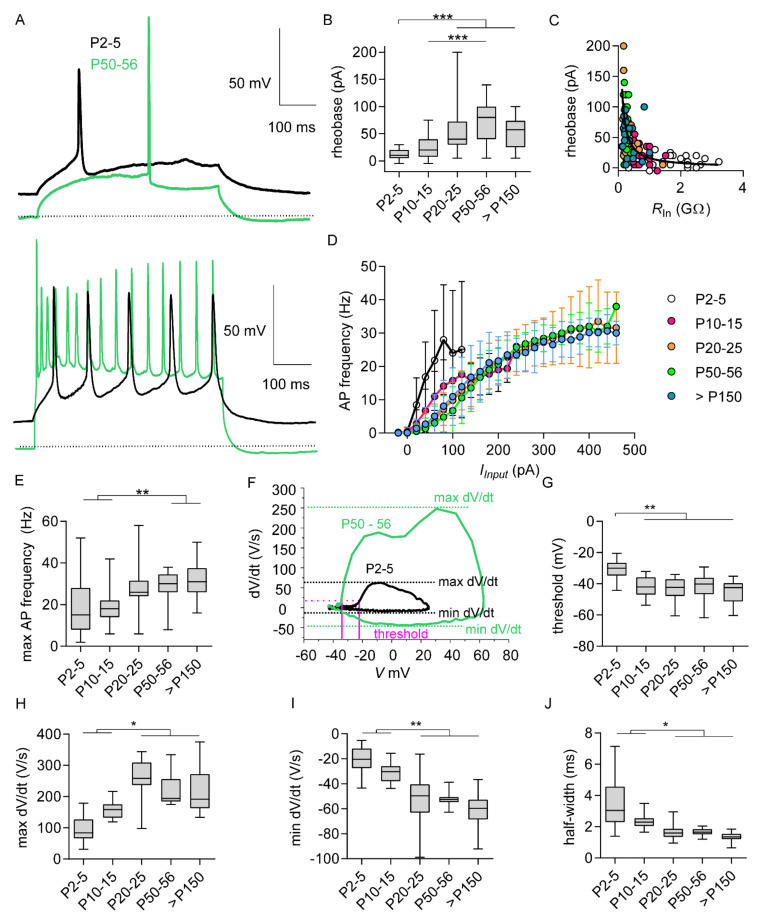
Development of intrinsic excitability and action potential firing in M1LV pyramidal neurons. (**A**) Upper panel: action potential (AP) elicited by rheobase currents (500 ms) from resting membrane potential in P2–5 neurons (black) and P50–56 neurons (green). Lower panel: maximal AP firing frequency at P2–5 (black) and P50–56 (green). (**B**) Rheobase significantly increased with age. (**C**) Relationship between rheobase and *R_In_* fitted by Ohm’s law (*I = V/R*), with *V* (16 mV, color code shown in G). (**D**) Input–output curve showing AP frequency elicited by current steps of 500 ms and of increasing amplitude (*I*_Input_). (**E**) Maximal AP firing frequency according to the age groups. (**F**) Phase plots of AP slope (dV/dt) against voltage (*V*) show P2–5 (black) to P50–56 (green) AP kinetics. Pink lines and dotted line indicate AP threshold (dV/dt > 20 V/s). Dashed lines highlight maximal and minimal slopes (max dV/dt and min dV/dt). (**G**)**.** AP threshold significantly decreased with age. (**H**–**I**) Peaks of the first derivative of voltage over time (dV/dt): max dV/dt (**H**), min dV/dt (**I**). (**J**) AP half-width. * *p* < 0.05; ** *p* < 0.01; *** *p* < 0.001; P2–5: *n* = 24; P10–15: *n* = 21; P20–25: *n* = 21; P50–56: *n* = 19; P > 150: *n* = 20 (more details in Table S4, Supplementary Materials).

Stepwise current injections of increasing amplitude (steps of 5 pA, 500 ms) were applied to induce progressive membrane depolarization, starting from *E*_rest_ (Figure 3A). The smallest current triggering AP (rheobase) increased with age (Figure 3B,C; Table S4, Supplementary Materials). Significant differences in rheobase were detected when comparing neurons of the first two postnatal weeks to neurons of the older groups. However, no significant differences were detected between age groups older than three weeks or between neurons of the first and second postnatal week (Table S4, Supplementary Materials). The age-related decrease in *R_In_* (Figure 1) may largely account for the increase in rheobase. Accordingly, the relationship between *R_In_* and rheobase in maturing neurons could be fitted by a hyperbole respecting Ohm’s law “*I = V/R*”, where *I* = rheobase, *R* = *R_In_*, and *V* (*R_In_* × rheobase) = 16 mV (Figure 3C). Functional maturation was also explored through the relationship between input and output with steps of increasing current amplitude (Δ = 20 pA, 500 ms), starting from *E*_rest_ (Figure 3A,D). Input–output gain, calculated as the differential of AP frequency over input step-current amplitude *d*AP frequency/*dI_i_*_nput_, decreased with age (Figure 3D; Table S4, Supplementary Materials). Significant differences were detected between neurons of the first postnatal week and those of some older age groups. However, no significant differences in gain were detected between age groups older than one week. Furthermore, the voltage at which maximal gain was reached increased with age (Table S4, Supplementary Materials). These data imply that cell growth and leakiness contribute to increased rheobase and affect input–output gain.

Other AP firing properties may be more tightly dependent on AIS maturation and elongation than rheobase and gain. For instance, maximal AP frequency increased with age (Figure 3E; Table S4, Supplementary Materials) and was significantly lower in neurons of the first two postnatal weeks compared to neurons of the two oldest groups. In contrast, no significant differences were detected between age groups older than three weeks, or between neurons of the first and second postnatal week (Table S4, Supplementary Materials).

Postnatal changes in AP threshold occurred as well, during the same period of maturation that applied to AP frequency and gain. Namely, the analysis of the first derivative of voltage over time (AP phase plot) revealed significant age-dependent AP threshold hyperpolarization by approximately 13 mV (AP threshold = 20V/s; Figure 3F,G; Table S4, Supplementary Materials). Significant differences in AP threshold were detected when comparing neurons of the first postnatal week to neurons of any older age group. In contrast, no significant differences were detected between age groups older than one week (Table S4, Supplementary Materials). In addition to AIS elongation, age-dependent hyperpolarization of the AP threshold might reflect changes in ion channels expressed by maturing M1LV neurons (Figure S1, Supplementary Materials). In turn, both factors may contribute to changes in AP kinetics.

To investigate this matter in more detail, kinetics of AP upstroke and repolarization were analyzed based on the maximum and minimum dV/dt. Our examination revealed that max dV/dt increased with age (Figure 3H; Table S4, Supplementary Materials), whereas min dV/dt decreased with age (Figure 3I; Table S4, Supplementary Materials). Once more, significant differences emerged comparing neurons of the first two postnatal weeks to neurons older than three weeks. In contrast, no significant differences were detected between age groups older than three weeks, or between neurons of the first and second postnatal week (Table S4, Supplementary Materials). Increased max dV/dt and decreased min dV/dt imply age-dependent acceleration of AP kinetics. Indeed, age-dependent acceleration of AP kinetics was confirmed by a significant decrease in AP half-width during maturation (Figure 3J; Table S4, Supplementary Materials). Significant differences were detected comparing neurons of the first two postnatal weeks to neurons older than three weeks. In contrast, no significant differences were detected between age groups older than three weeks, or between neurons of the first and second postnatal week (Table S4, Supplementary Materials). In summary, significant changes of AP threshold distinguished two age-categories during maturation, i.e., before and after P5. Furthermore, the age-related acceleration of AP kinetics pointed to an additional maturation milestone occurring at a slightly later time point, i.e., by the end of the third postnatal week.

### 2.3. Initial Segment Component and Somatodendritic Component Mirror Prominent AIS Elongation

**Figure 4:**
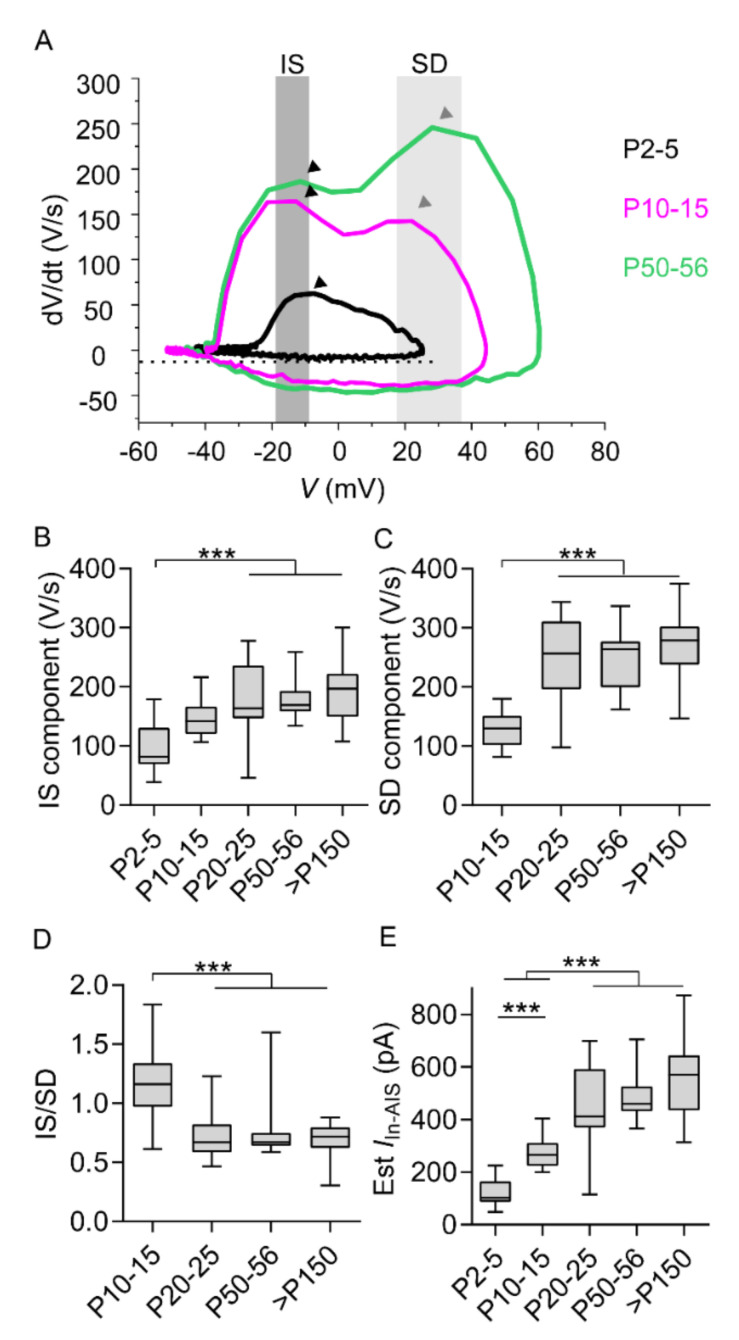
Development of AIS (IS) and somatodendritic (SD) components. (**A**) Phase plots show slope of AP (dV/dt) at P2–5 (black), P10–15 (pink), and P50–55 (green). Shaded areas and arrowheads highlight the maximum-slope peaks of phase 1 (dark gray) and phase 2 (light gray). (**B**,**C**) IS component (**B**) and SD component (**C**). (**D**) Ratio between IS and SD components. (**E**) Estimated inward currents at the AIS (Est *I*_in-AIS_), according to IS amplitude and AIS capacitance (I = C × dV/dt). *** *p* < 0.001 P2–5: *n* = 24; P10–15: *n* = 21; P20–25: *n* = 21; P50–56: *n* = 19; P > 150: *n* = 20 (more details in Table S5, Supplementary Materials).

To analyze further features of AP maturation, the relative maximum of dV/dt (highlighted by gray bands in Figure 4A) was used to determine the AIS component (IS) of each AP phase plot. In addition, the absolute maximum of dV/dt (highlighted by gray bands in Figure 4A) was used to determine the somatodendritic component (SD). Furthermore, IS and SD components were compared among age groups (Figure 4; Table S5, Supplementary Materials). At P2–5, the AP displayed one component, supposedly attributed to the AIS (Figure 4A,B; Table S5, Supplementary Materials). Significant differences were detected between neurons of the first postnatal week and neurons older than three weeks. In contrast, no significant differences were detected between age groups older than three weeks, or between neurons of the first and second postnatal week (Figure 4A,B; Table S5, Supplementary Materials).

After the first postnatal week, APs were characterized by two phases (Figure 4A), including both the IS and the SD component. The SD component appeared consistently in neurons older than P10 and increased with age (Figure 4A,C; Table S5, Supplementary Materials). Significant differences in the SD component and the ratio between IS and SD components (Figure 4D) were detected when neurons of the second postnatal week were compared to neurons of any older age group. In contrast, no significant differences in SD component were detected between age groups older than three weeks.

Average AIS length and diameter (Figure 2; Table S2, Supplementary Materials) for each age group allowed estimating the AIS capacitance (*C*_AIS_). Furthermore, since the IS component is mediated by currents at the AIS [23,24], the product of IS component and AIS capacitance allowed gauging age-related changes in inward current amplitude at the AIS (estimated (Est) *I*_In-AIS_ = *C*_AIS_ × IS component). Being the product of an increasing AIS capacitance and an increasing IS component (Figure 4A–C), the amplitude of Est *I*_In-AIS_ increased with age as well (Figure 4E; Table S5, Supplementary Materials). Significant differences in amplitude of Est *I*_In-AIS_ were detected comparing neurons of the first two postnatal weeks to neurons older than three weeks. Furthermore, significant differences were detected comparing neurons of the first postnatal week with neurons of the second postnatal week. In contrast, no significant differences were detected between age groups older than three weeks (Figure 4E, Table S5, Supplementary Materials).

### 2.4. Development of AP After-Hyperpolarization and AP After-Depolarization Endure Throughout Adulthood

**Figure 5:**
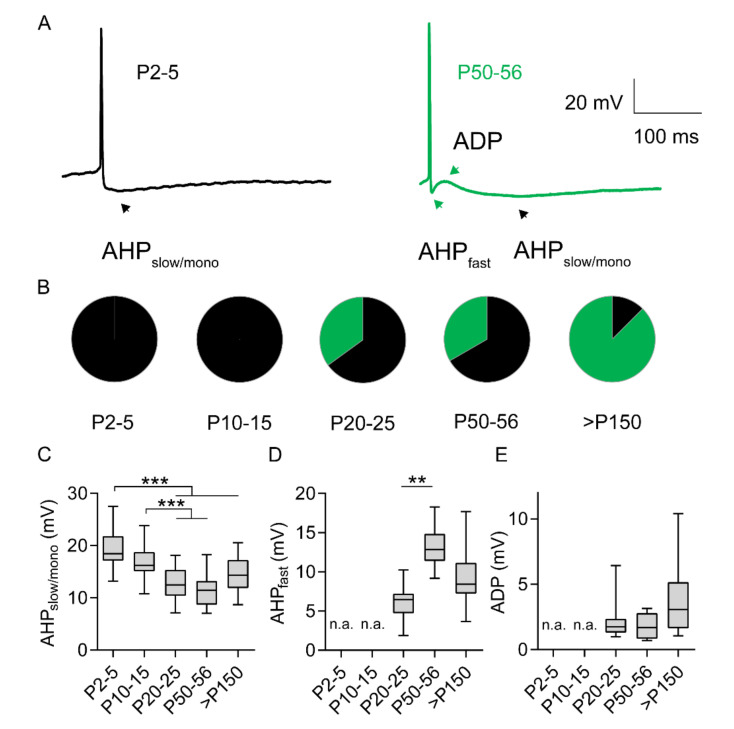
Development of after-hyperpolarization (AHP) and after-depolarization (ADP). (**A**) Representative APs displaying one-phase AHP_slow/mono_ (black) and three-phase AHP_fast_-ADP-AHP_slow/mono_ (green). (**B**) Proportion of neurons displaying AHP_slow/mono_ (black) and AHP_fast_-ADP-AHP_slow/mono_ (green) per age group. (**C**) Amplitude of AHP_slow/mono_. (**D**) Amplitude of AHP_fast_. **E.** ADP amplitude. n.a. = not applicable. ** *p* < 0.01; *** *p* < 0.001. *n* mono-phasic/*n* tri-phasic: P2–5 = 21/0; P10–15 = 19/0; P20–25 = 20/7; P50–56 = 18/6; P > 150 = 16/14 (more details in Table S6, Supplementary Materials).

As further evidence of physiological maturation of M1LV neurons, the shape of AP after-hyperpolarization (AHP) changed with age (Figure 5A,B). Qualitative differences marked an age-dependent group repartition before and after the third postnatal week. Specifically, slow mono-phasic AHP events (AHP_slow/mono_) were detected consistently in neurons from rats younger than two weeks (Figure 5A,B; Table S6, Supplementary Materials). Conversely, neurons from rats older than three weeks displayed either mono-phasic or tri-phasic events. In tri-phasic events, fast AHP (AHP_fast_) was followed by after-depolarization (ADP) and, eventually, by AHP_slow/mono_ (Figure 5A). The proportion of neurons displaying tri-phasic events increased with age (Figure 5B; Table S6, Supplementary Materials).

The relative amplitude of AHP and ADP was quantified in reference to AP threshold. Accordingly, the amplitude of AHP_slow/mono_ decreased with age. Significant differences were detected comparing neurons of the first two postnatal weeks to neurons of groups older than three weeks. In contrast, no significant differences were detected between age groups older than three weeks (Figure 5A,C; Table S6, Supplementary Materials). In adult neurons, the amplitude of AHP_fast_ increased significantly from the third postnatal week to the second month and decreased slightly, but not significantly (*p* = 0.06), from the second to the fifth month (Figure 5A,D; Table S6, Supplementary Materials). Furthermore, in adult neurons, the amplitude of ADP did not change significantly with age (Figure 5A,E; Table S6, Supplementary Materials). Age-related differences of AHP_fast_ implied some degree of functional maturation in neurons older than three weeks. In this study, the adjustment of AHP_fast_ in adult rats represents the latest event of intrinsic functional maturation detected in M1LV neurons, and the only event of significant functional maturation was detected after P20.

## 3. Discussion

In rodents, neurons in the brain undergo significant postnatal growth and maturation. During this process, increasing size and complexity dictate changes of biophysical cell membrane properties. The membrane capacitance *C*_m_ reflects size. In M1LV neurons, *C*_m_ increased dramatically in the first postnatal week, outlining the period of most prominent growth. While *C*_m_ increased, the input resistance (*R_In_*), which is inversely related to passive transmembrane electric leakage, decreased. Thus, M1LV neurons became electrically leakier upon maturation, maintaining a relatively constant proportion between cell size and leakage.

Changes in *R_In_* are commonly observed in maturing neurons [25,26,27,28]. The process affects intrinsic excitability due to the inverse relationship between *R_In_* and rheobase (minimum current that elicits APs). According to Ohm’s law, the relationship between *R_In_* and rheobase can be expressed as

$$\mathrm{rheobase}\;=\frac{V}{R_{In}}$$

where *R_In_* is an independent variable and rheobase is a dependent variable. If the voltage *V* is assumed constant, the relationship between rheobase and *R_In_* can be outlined by a hyperbole, as shown in Figure 3. Notably, data from each sample distributed along a unique hyperbole even though they belonged to different age groups, implying that (1) the age-related increase of rheobase in M1LV neurons is largely justified by changing *R_In_* (as explained by Ohm’s law), and (2) the voltage *V,* equal to depolarization at rheobase, should be assumed constant across all age groups. To validate the latter assumption, depolarization at rheobase could be estimated empirically as the difference between *E*_rest_ and AP threshold. In M1LV neurons, neither *E*_rest_ nor AP threshold remained constant over age. However, both parameters varied synchronously and to the same extent. Therefore, changes of *E*_rest_ and AP threshold had little effect on the rheobase in our experimental settings, and depolarization at rheobase was indeed constant across all age groups.

The equivalence and synchronicity of age-related changes in AP threshold and *E*_rest_ was striking. Whether the two processes are mediated by the same mechanism remains speculative. Hyperpolarization of *E*_rest_, common in maturing neurons, might involve tonic currents at hyperpolarized potentials [29,30,31]. On the other hand, AP threshold may be controlled by AIS maturation. Additionally, AP firing properties could be influenced by developmental changes in ion channel expression (see Appendix A), also documented in previous works [32,33,34,35,36]. Changes in IS and SD components may reflect the maturation of AIS-related mechanisms controlling the AP upstroke [24]. All these physiological changes occurred at the same time and may concur with hyperpolarization of the AP threshold. However, the influence of different sodium channel isoforms on the voltage sensitivity of the AP threshold is debatable [23]. Conversely, IS component maturation, SD component maturation, and prominent AIS elongation adequately explain AP threshold hyperpolarization as supported by *in silico* models [12,13].

AP firing frequency and AP upstroke slope (max dV/dt) constitute further features of AP firing influenced by AIS maturation. In M1LV neurons, age-related changes of both parameters occurred in the period of most prominent AIS elongation. These changes aligned with predicted effects of AIS elongation and likely contributed to optimal input–output conversion in growing neurons, according to the known relationship between optimal AIS length and cell size [12,13].

Our observations suggest that postnatal AIS elongation largely contributes to the maturation of several output properties in growing M1LV neurons. In sensory cortices, phases of postnatal AIS remodeling are associated with phasic adaptation of intrinsic neuronal excitability [15,17,18,37]. In contrast, the AIS of M1LV neurons underwent continuous elongation without phasic remodeling of intrinsic excitability. In light of the continuous process of morphological and functional maturation of M1LV neurons, our study implies that the most prominent AIS elongation associated with the most significant functional maturation soon after birth. Conversely, small rates of AIS elongation reflected negligible functional maturation in adulthood.

In conclusion, our findings draw a connection between AIS elongation and postnatal cell growth, in which AIS length relates to cell size. Our study focused on M1LV as a whole population despite the heterogeneous connectivity of M1LV neurons [38], which may slightly affect the course of functional maturation, thereby contributing to neuron-to-neuron variability within each age group. Thus, subtle aspects of M1LV neuron maturation might emerge from a subtype analysis based on the output-specific connectivity. The characterization of the continuous process of AIS elongation and functional cellular maturation under unchallenged physiological conditions establishes the foundation for understanding the impact of motor learning and pathological conditions in this pivotal cortical region.

## 4. Materials and Methods

### 4.1. Use of Animals for Experiments

Experiments were performed in agreement with “Directive 2010/63/EU of the European Parliament and of the Council of 22 September 2010 on the protection of animals used for scientific purposes” and were approved by Austrian and German animal care authorities: protocol numbers BMWFW-66.019/0004-WF/V/3b/2017 approved on 26th January 2017 by the Austrian Federal Ministry for Education, Science and Research and I-18/07, approved on 2nd February 2018 by the German Ministry of Science, Research and Arts, Karlsruhe, Germany.

### 4.2. Immunofluorescence and Image Analysis

Animals in age groups up to P7 were sacrificed by cervical dislocation after brief isoflurane inhalation. Brains were removed and immersion-fixed in 2% paraformaldehyde (PFA; pH 7.4) for 4 h. Animals P10 and older received terminal anesthesia by intraperitoneal injection of ketamine (273 mg per kg body weight), xylazine (7.1 mg per kg body weight), and acepromazine (0.625 mg per kg body weight), verified by complete loss of paw and corneal reflexes. Rats were then transcardially perfused with 0.9% NaCl for 5 min, followed by 0.1 M phosphate-buffered 2% PFA for 10 min. Dissected brains were washed in 0.1 M phosphate buffer and transferred into 0.1 M phosphate buffered 30% sucrose solution (pH 7.4) at 4 °C for at least 72 h. Tissue was trimmed to a block including M1 from caudal to rostral sections and embedded in Tissue Tek (Sakura Finetek, Staufen, Germany). Brains of animals up to P10 were cut coronally using a cryostat (Microm HM550, ThermoFisher, Dreieich, Germany) at 20 µm directly onto glass slides. Brains from animals P15 and older were cut in coronal sections (40 µm) and processed for free-floating immunostaining as outlined below.

Sections were incubated in blocking buffer (1% bovine serum albumin (BSA), 0.2% fish skin gelatin, 0.1% Triton in phosphate-buffered saline (PBS)) for at least 60 min and subsequently incubated in primary antibodies overnight. Primary antibodies used were rabbit anti-βIV-spectrin (1:1000, self-made [15]) and guinea pig anti-NeuN (1:750, Merck Millipore, Darmstadt, Germany; Cat# ABN90, RRID:AB_11205592). After washing in PBS, the slices were incubated for at least 90 min in secondary antibodies. Secondary antibodies used were donkey anti-rabbit Alexa 488 and 568, donkey anti-guinea-pig Alexa 568 and 647, and donkey anti-mouse Alexa 647 (all 1:1000 except Alexa 647 1:500, Molecular Probes, ThermoFisher, Dreieich, Germany; Cat# A-21206, RRID:AB_141708, Cat# A-11075, RRID:AB_141954, Cat# A-21450, RRID:AB_141882, Cat# A-31571, RRID:AB_162542). For preservation of immunofluorescence, slices were mounted in Roti-Mount FluorCare (Carl Roth, Karlsruhe, Germany). Fluorescence images were acquired using a Nikon C2 confocal microscope with 40× and 60× objectives (numerical aperture 1.4). AIS length was determined using the Python-based, self-written software AISuite as previously described [39,40,41]. The AISuite software is available via github.com/jhnnsrs/aisuite2. This tool extends the well-established method of defining proximal distal AIS boundaries as points where a predefined fluorescence threshold, which is relative to the maximum fluorescence intensity along a line drawn over an individual AIS, is surpassed [10]. In our study, the threshold was adjusted depending on the individual staining and ranged from 10–30% of maximum fluorescence intensity. Representative immunofluorescence images in figures were enhanced for contrast and brightness using Photoshop CS4 (Adobe Software, San Jose, CA, USA).

### 4.3. Electrophysiology

Rats for electrophysiological analysis were anesthetized with isoflurane and decapitated. Brains were dissected while submerged in chilled artificial cerebrospinal fluid (ACSF). Chilled high-sucrose ACSF was used for slice preparation and contained (in mM) 206.0 sucrose, 25 NaCO_3_, 25 glucose, 1.0 CaCl_2_, 3.0 MgCl_2_, 2.5 KCl, and 1.25 NaH_2_PO_4_ (osmolarity = 309 mOsm) [42]. Coronal sections were sliced with a Leica VT1200s microtome at a thickness of 250 µm while submerged in chilled ACSF and transferred into a storage chamber, where they were submerged in room temperature ACSF. ACSF used for slice storage and measurements of brain slices contained (in mM) 134 NaCl, 26 NaHCO_3_, 25.0 glucose, 2.0 CaCl_2_, 1.0 MgCl_2_, 2.4 KCl, and 1.25 NaH_2_PO_4_; pH was balanced to 7.4, using a mix of CO_2_/O_2_ (95/5%) (osmolarity = 315 mOsm). The set-up for electrophysiological recordings consisted of an Olympus upright microscope equipped with motorized micromanipulators and stage (Scientifica, Uckfield, UK). During recording, brain slices were held in a chamber with a volume of 0.5–1.0 mL of ACSF and a flow of ACSF equal to 0.5–1.0 mL/min. Patch pipettes had a resistance of 3–4.5 MΩ and allowed achieving comparable R_S_ in different age groups. The intracellular pipette solution contained (in mM) 135 K-gluconate, 4 KCl, 10 HEPES, 10 Na-phosphocreatine, 4 ATP-Mg, and 0.3 GTP-Na [42]. The pH was adjusted to 7.25 (osmolarity = 300 mOsm). Osmolarity was measured with a Vapro (Wescor, South Logan, Utah, USA) osmometer.

Cells of upper layer V in M1 were targeted in this study. Recordings were acquired with a HEKA amplifier (HEKA, Lambrecht, Germany) at 10 KHz, filtered at 2 KHz, and analyzed with FitMaster (HEKA), Origin (OriginLab, Northampton, MA, USA) and Prism 8 (GraphPad, San Diego, CA, USA) software. Rheobase was determined with current clamp protocols, consisting of consecutive 500 ms-long hyperpolarizing and depolarizing steps from resting membrane potential (E_rest_). Hyperpolarizing steps started at −20 pA, adding 5 pA to each consecutive step, until rheobase was reached. To determine the relationship between input current and action potential frequency, larger steps were used (20 pA), starting at −20 pA and up to 250–300 pA. During inter-step intervals, the membrane was kept at resting potential for 500 ms. Voltage-clamp protocols used to determine the current–voltage relationship of voltage-gated currents consisted of depolarizing steps (20 mV) of 500 ms, from −90 mV to +20 mV. Between steps, voltage was held at −70 mV for 4 s.

### 4.4. Experimental Design and Statistical Analysis

Comparison of AIS length in different age groups (Figure 2) was performed using measurements carried out on 4–6 animals of either sex per age group, providing at least 600 AIS per age group in total (see Table S2, Supplementary Materials). Sample size was defined according to previous reports [15,17] and subsequent power analysis. Individual rats were considered as biological replicates. For each biological replicate, individual AIS length was averaged as technical replicate. Statistical analysis was carried out according to the number of biological replicates, and data are presented as mean ± SD.

For comparing physiological properties among age groups, we pre-designated a sample size of c.a. 20 neurons per age group (see Tables S3–S7, Supplementary Materials). Five age groups evenly spaced across the time considered in the morphological analysis (P0 to P150) were designated, with brackets of 3–5 days. In light of the age-related AIS elongation rate, the oldest group involved an open age bracket (P > 150 = P166 ± 30; average ± SD).

Single recordings from individual neurons were considered as biological replicates and used in the statistical analysis as independent samples. Independent samples were acquired from three or more rats within each age-bracket (detailed sample size: P2–5 = 4 rats, 24 neurons; P10–15 = 5 rats, 21 neurons; P20–25 = 3 rats, 21 neurons; P50–56 = 5 rats, 19 neurons; P > 150 = 9 rats, 20 neurons).

In all experiments, each group of data was firstly tested for normality of distribution before testing for statistical significance in multiple comparisons, and populations were analyzed with one-way ANOVA or Kruskal–Wallis test, accordingly. Age-related differences implied a significance in the multiple comparison test. Multiple comparison tests were followed by a post hoc test (Bonferroni’s or Dunn’s multiple comparison test), where differences between individual age groups were tested in permutation. Significant differences between groups are indicated by asterisks in figures (* *p* < 0.05; ** *p* < 0.01; *** *p* < 0.001). In the box plots, data are reported as median, inter-quartile, and min–max distribution. In *XY* plots, data are represented as mean ± SD (Figure 2C and Figure 3D; Figure S1B,E,G, Supplementary Materials) or as single samples (scatter plots), color-coded by age (Figure 1C and Figure 3C; Figure S1F, Supplementary Materials). Sample sizes and *p*-values for every statistical comparison are detailed in all tables in the Supplementary Materials, next to each respective mean and SD.

## Supplementary Materials

Supplementary materials can be found at https://www.mdpi.com/1422-0067/21/17/6101/s1. Figure S1: Development of voltage-activated currents, Tables S1–S7: Statistical analysis.

## Abbreviations

AIS	Axon initial segment
AP	Action potential
M1	Primary motor cortex
M1LV	Layer V of the primary motor cortex
*C*_m_	Membrane capacitance
*R*_in_	Input resistance
*E*_rest_	Resting membrane potential
V	Voltage
R	Resistance
I	Current
*I*_max_	Maximal current
*I*_mem_	Steady-state membrane leak current
I_In_	Inward current
I_In_V_Half_	Voltage of half-maximal activation of the inward current
dV/dt	Derivative of voltage over time
τ	Decay time-constant of maximal current
IS	Axon initial segment component
SD	Somatodendritic component
Est*I*_In-AIS_	Estimated current at the axon initial segment
*C*_AIS_	Capacitance of the axon initial segment
ADP	After-depolarization
AHP	After-hyperpolarization
AHP_fast_	Fast component of the after-hyperpolarization
AHP_slow/mono_	Slow or single component of the after-hyperpolarization

## Appendix A

### Development of Voltage-Gated Currents in MILV Neurons

Developmental maturation of voltage-activated inward currents partakes in the age-dependent physiological development of M1LV neurons. To estimate the timing of the process, voltage-activated inward and outward currents were measured in voltage-clamp experiments, progressively depolarizing neurons from a holding potential of −70 mV (Δ = 20 mV, 500 ms; Figure S1, Supplementary Materials).

The peak amplitude of inward currents (*I*_In_) increased with age (Figure S1A–C, Table S7, Supplementary Materials). Significant differences were detected comparing neurons of the first postnatal week to neurons of any older age group (Figure S1A–C, Supplementary Materials). Nevertheless, increased *I*_In_ amplitude was proportional to *C*_m_, resulting in comparable current densities among age groups. More interestingly, the voltage sensitivity of *I*_In_ increased with age as revealed by hyperpolarization of the half-maximal activation of *I*_In_ (*I*_In_*V_Half_*; Figure S1D,E, Table S7, Supplementary Materials). Significant differences in *I*_In_*V_Half_* were detected upon comparison of neurons of the first postnatal week to neurons of any older age group. However, no significant differences were detected between age groups older than one week. Both AP threshold and *I*_In_*V_Half_* underwent a shift of approximately 13 mV and, thus, changes in the values of *I*_In_*V_Half_* paralleled changes in the AP threshold. This applies to the timing and magnitude as well.

These data do not imply a causal relationship between changing *I*_In_*V_Half_* and AP threshold. In the absence of pharmacological dissection of current components, and in view of the large current amplitudes limiting the quality of voltage clamp, this observation remains a qualitative assessment. Furthermore, whole-cell measurements do not represent the sole changes of currents at the AIS. However, no significant correlation was found between peak amplitude and *I*_In_*V_Half_* (Figure S1F, Supplementary Materials). Furthermore, *I*_In_ currents peaked before significant outward current activation *I*_Out_ (Figure S1G, Supplementary Materials). Thus, the observed voltage shift of *I*_In_*V_Half_* may actually reflect the change in voltage-gated inward currents in M1LV neurons over age. The Supplementary Materials, therefore, hint toward a main time point of maturation in whole-cell voltage-gated currents that accompanies the early process of AIS maturation, thus affecting properties of AP firing.

## References

[1] Bakkum D.J., Obien M.E.J., Radivojevic M., Jäckel D., Frey U., Takahashi H., Hierlemann A. (2018). The axon initial segment is the dominant contributor to the neuron’ s extracellular electrical potential landscape. Adv. Biosys.

[2] Hodgkin A.L., Huxley F. (1952). A quantitative description of membrane current and its application to conduction and excitation in nerve. J. Physiol..

[3] Kole M.H.P., Ilschner S.U., Kampa B.M., Williams S.R., Ruben P.C., Stuart G.J. (2008). Action potential generation requires a high sodium channel density in the axon initial segment. Nat. Neurosci..

[4] Bender K.J., Trussell L.O. (2012). The physiology of the axon initial segment. Annu. Rev. Neurosci..

[5] Kole M.H.P., Stuart G.J. (2012). Signal processing in the axon initial segment. Neuron.

[6] Susuki K., Kuba H. (2016). Activity-dependent regulation of excitable axonal domains. J. Physiol. Sci..

[7] Petersen A.V., Cotel F., Perrier J.F. (2017). Plasticity of the axon initial segment: fast and slow processes with multiple functional roles. Neuroscientist.

[8] Jamann N., Jordan M., Engelhardt M. (2018). Activity-dependent axonal plasticity in sensory systems. Neuroscience.

[9] Wefelmeyer W., Puhl C.J., Burrone J. (2016). Homeostatic plasticity of subcellular neuronal structures: from inputs to outputs. Trends Neurosci..

[10] Grubb M.S., Burrone J. (2010). Activity-dependent relocation of the axon initial segment fine-tunes neuronal excitability. Nature.

[11] Kuba H., Oichi Y., Ohmori H. (2010). Presynaptic activity regulates Na 1 channel distribution at the axon initial segment. Nature.

[12] Kole M.H., Brette R. (2018). The electrical significance of axon location diversity. Curr. Opin. Neurobiol..

[13] Goethals S., Brette R. (2020). Theoretical relation between axon initial segment geometry and excitability. Elife.

[14] Kast R.J., Levitt P. (2019). Progress in Neurobiology Precision in the development of neocortical architecture: From progenitors to cortical networks. Prog. Neurobiol..

[15] Schlüter A., Del Turco D., Deller T., Gutzmann A., Schultz C., Engelhardt M. (2017). Structural plasticity of synaptopodin in the axon initial segment during visual cortex development. Cereb. Cortex.

[16] Kuba H., Adachi R., Ohmori H. (2014). Activity-dependent and activity-independent development of the axon initial segment. J. Neurosci..

[17] Gutzmann A., Ergül N., Grossmann R., Schultz C., Wahle P., Engelhardt M. (2014). A period of structural plasticity at the axon initial segment in developing visual cortex. Front. Neuroanat..

[18] Kim E.J., Feng C., Santamaria F., Kim J.H., Inserm U. (2019). Impact of auditory experience on the structural plasticity of the ais in the mouse brainstem throughout the lifespan. Front. Cell. Neurosci..

[19] Dooley J.C., Blumberg M.S. (2018). Developmental ’awakening’ of primary motor cortex to the sensory consequences of movement. Elife.

[20] Weiler N., Wood L., Yu J., Solla S.A., Shepherd G.M.G. (2008). Top-down laminar organization of the excitatory network in motor cortex. Nat. Neurosci..

[21] Tjia M., Yu X., Jammu L.S., Lu J., Zuo Y. (2017). Pyramidal neurons in different cortical layers exhibit distinct dynamics and plasticity of apical dendritic spines. Front. Neural Circuits.

[22] Young N.A., Vuong J., Teskey G.C., Young N.A., Vuong J., Teskey G.C. (2012). Development of motor maps in rats and their modulation by experience. J. Neurophysiol..

[23] Katz E., Stoler O., Scheller A., Khrapunsky Y., Goebbels S., Kirchhoff F. (2018). Role of sodium channel subtype in action potential generation by neocortical pyramidal neurons. Proc. Natl. Acad. Sci. USA.

[24] Lazarov E., Dannemeyer M., Feulner B., Enderlein J., Gutnick M.J., Wolf F., Neef A. (2018). An axon initial segment is required for temporal precision in action potential encoding by neuronal populations. Sci. Adv..

[25] Benedetti B., Dannehl D., König R., Coviello S., Kreutzer C., Zaunmair P., Jakubecova D., Weiger T.M., Aigner L., Nacher J. (2019). Functional Integration of Neuronal Precursors in the Adult Murine Piriform Cortex. Cereb. Cortex.

[26] Schmidt-Hieber C., Jonas P., Bischofberger J. (2004). Enhanced synaptic plasticity in newly generated granule cells of the adult hippocampus. Nature.

[27] Liu Y.B., Lio P.A., Pasternak J.F., Trommer B.L. (1996). Developmental changes in membrane properties and postsynaptic currents of granule cells in rat dentate gyrus. J. Neurophysiol..

[28] Lodge M., Bischofberger J. (2019). Synaptic properties of newly generated granule cells support sparse coding in the adult hippocampus. Behav. Brain Res..

[29] Suwabe T., Mistretta C.M., Krull C., Bradley R.M. (2011). Pre-and postnatal differences in membrane, action potential, and ion channel properties of rostral nucleus of the solitary tract neurons. J. Neurophysiol..

[30] Bao H., Bradley M.R., Mistretta M.C. (1995). Development of intrinsic electrophysiological properties in neurons from the gustatory region of rat nucleus of solitary tract. Dev. Brain Res..

[31] Hu W., Bean B.P. (2018). Differential control of axonal and somatic resting potential by voltage-dependent conductances in cortical layer 5 pyramidal neurons. Neuron.

[32] Beckh S., Noda M., Lübbert H., Numa S. (1989). Differential regulation of three sodium channel messenger RNAs in the rat central nervous system during development. EMBO J..

[33] Yarowsky P.J., Krueger B.K., Erik Olson C., Clevinger E.C., Koos R.D. (1991). Brain and heart sodium channel subtype mRNA expression in rat cerebral cortex. Proc. Natl. Acad. Sci. USA.

[34] Boiko T., Rasband M.N., Levinson S.R., Caldwell J.H., Mandel G., Trimmer J.S., Matthews G. (2001). Compact myelin dictates the differential targeting of two sodium channel isoforms in the same axon. Neuron.

[35] Boiko T., Van Wart A., CaldWell J.H., Levinson S.R., Trimmer J.S., Matthews G. (2003). Functional specialization of the axon initial segment by isoform-specific sodium channel targeting. J. Neurosci..

[36] Alberto Sánchez-Aguilera G.M., Colino A., Vicente-Torres M.Á. (2020). Development of action potential waveform in hippocampal CA1 pyramidal neurons. Neuroscience.

[37] Jamann N., Dannehl D., Wagener R., Corcelli C., Schultz C., Staiger J., Kole M.H.P., Engelhardt M. (2020). Sensory input drives rapid homeostatic scaling of the axon initial segment in mouse barrel cortex. bioRxiv.

[38] Suter B.A., Migliore M., Shepherd G.M.G. (2013). Intrinsic electrophysiology of mouse corticospinal neurons: A class-specific triad of spike-related properties. Cereb. Cortex.

[39] Höfflin F., Jack A., Riedel C., Mack-Bucher J., Roos J., Corcelli C., Schultz C., Wahle P., Engelhardt M. (2017). Heterogeneity of the axon initial segment in interneurons and pyramidal cells of rodent visual cortex. Front. Cell. Neurosci..

[40] Schlüter A., Rossberger S., Dannehl D., Janssen J.M., Vorwald S., Hanne J., Schultz C., Mauceri D., Engelhardt M. (2019). Dynamic Regulation of Synaptopodin and the Axon Initial Segment in Retinal Ganglion Cells During Postnatal Development. Front. Cell. Neurosci..

[41] Ernst L., Darschnik S., Roos J., González-Gómez M., Beemelmans C., Beemelmans C., Engelhardt M., Meyer G., Wahle P. (2018). Fast prenatal development of the NPY neuron system in the neocortex of the European wild boar, Sus scrofa. Brain Struct. Funct..

[42] Van Aerde K.I., Feldmeyer D. (2015). Morphological and physiological characterization of pyramidal neuron subtypes in rat medial prefrontal cortex. Cereb. Cortex.

