# Supplementary material for: Structural and Functional Maturation of Rat Primary Motor Cortex Layer V Neurons
Source: Int J Mol Sci. 2020 Aug 24;21(17):6101. doi: 10.3390/ijms21176101 (PMC7503395; doi:10.3390/ijms21176101)
Supplement: Supplementary file 1 [file ijms-21-06101-s001.pdf]

Supplementary Table 1

	**P2-5**<br>**(n = 24)**	**P10-15**<br>**(n = 21)**	**P20-25**<br>**(n = 21)**	**P50-56**<br>**(n = 19)**	**P > 150**<br>**(n = 20)**	**oneway ANOVA**<br>**+**<br>***post-hoc test***
C_m (nF)	0.04<br>± 0.02	0.10<br>± 0.04	0.16<br>± 0.05	0.11<br>± 0.02	0.14<br>± 0.07	Kruskal-Wallis test: p < 0.0001<br>Dunn's multiple comparisons test:<br>P2-5 vs. P10-15: p =0.0005<br>P2-5 vs. P20-25: p < 0.0001<br>P2-5 vs. P50-56: p = 0.0001<br>P2-5 vs. P > 150: p < 0.0001<br>P10-15 vs. P 20-25: p = 0.0215<br>P10-15 vs. P 50-56: p > 0.9999<br>P10-15 vs. P > 150: p > 0.9999<br>P20-25 vs. P 50-56: p = 0.1059<br>P20-25 vs. P > 150: p > 0.9999<br>P50-56 vs. P 150: p > 0.9999
R_{In} (GΩ)	1.61<br>± 0.88	0.74<br>± 0.31	0.37<br>± 0.28	0.25<br>± 0.07	0.36<br>± 0.24	Kruskal-Wallis test: p < 0.0001<br>Dunn's multiple comparisons test:<br>P2-5 vs. P10-15: p > 0.9999<br>P2-5 vs. P20-25: p < 0.0001<br>P2-5 vs. P50-56: < 0.0001<br>P2-5 vs. P > 150: < 0.0001<br>P10-15 vs. P 20-25: p = 0.0098<br>P10-15 vs. P 50-56: p = 0.0001<br>P10-15 vs. P > 150: p = 0.0074<br>P20-25 vs. P 50-56: p > 0.9999<br>P20-25 vs. P > 150: p > 0.9999<br>P50-56 vs. P 150: p > 0.9999
E_{rest} (mV)	-58<br>± 9	-68<br>± 9	-70<br>± 6	-72<br>± 10	-72<br>± 10	ANOVA: p < 0.0001<br>Bonferroni's multiple comparisons test<br>P2-5 vs. P10-15: p =0.0007<br>P2-5 vs. P20-25: p < 0.0001<br>P2-5 vs. P50-56: < 0.0001<br>P2-5 vs. P > 150: < 0.0001<br>P10-15 vs. P 20-25: p = 0.9792<br>P10-15 vs. P 50-56: p = 0.7538<br>P10-15 vs. P > 150: p = 0.7725<br>P20-25 vs. P 50-56: p = 0.9664<br>P20-25 vs. P > 150: p = 0.9736

						P50-56 vs. P 150: p > 0.9999

Development of passive membrane properties in M1LV neurons. Capacitance (C_m), input resistance (R_{in}), and resting membrane potential (E_{rest}) are reported as average ± standard deviation. P values for multiple comparison and post-hoc test are reported for each paired comparison. The statistical tests used for normally distributed samples were one-way ANOVA and Bonferroni's multiple comparisons test (A. + Bonferroni). Non-normally distributed samples were compared with the Kruskal-Wallis test and Dunn's multiple comparisons test (K-W + Dunn's).

<table>
<tr><th colspan="10">Supplementary Table 2</th></tr>
<tr><th></th><th>P1</th><th>P3</th><th>P5</th><th>P7</th><th>P10</th><th>P15</th><th>P28</th><th>P55</th><th>P150</th></tr>
<tr><td>AIS length (µm)</td><td>(n = 4)
13.5
± 0.8</td><td>(n = 6)
15.6
± 0.4</td><td>(n = 6)
16.5
± 0.4</td><td>(n = 6)
17.5
± 0.8</td><td>(n = 6)
20.6
± 1.4</td><td>(n = 6)
22.4
± 2.1</td><td>(n = 6)
24.4
± 1.7</td><td>(n = 6)
26.5
± 1.2</td><td>(n = 6)
28.3
± 0.5</td></tr>
<tr><td>AIS prox. diameter (µm)</td><td colspan="3">(n = 3)
1.34
± 0.09</td><td></td><td colspan="2">(n = 3)
1.56
± 0.15</td><td colspan="3">(n = 3)
2.04
± 0.15</td></tr>
<tr><td>AIS dist. diameter (µm)</td><td colspan="3">0.99
± 0.05</td><td></td><td colspan="2">1.07
± 0.15</td><td colspan="3">1.08
± 0.04</td></tr>
<tr><td>AIS distance from soma (µm)</td><td colspan="3">1.79
± 0.30</td><td></td><td colspan="2">1.29
± 0.21</td><td colspan="3">1.07
± 0.21</td></tr>
</table>

Developmental AIS elongation in M1LV pyramidal neurons. AIS length, proximal and distal diameter, and distance from soma for M1LV neurons are reported as average ± standard deviation. Number of animals (n) used for the analysis of each age group are reported below headers, indicating each respective age group (at least 100 AIS/animal).

Supplementary Table 3

ANOVA p < 0.0001

Bonferroni's multiple comparisons test:

P1 vs. P3:<br>*p* = 0.2014<br>P1 vs. P5<br>*p* = 0.0143<br>P1 vs. P7:<br>*p* = 0.0004<br>P1 vs. P10:<br>*p* <0.0001<br>P1 vs. P15:<br>*p* <0.0001<br>P1 vs. P28:<br>*p* <0.0001<br>P1 vs. P55:<br>*p* <0.0001<br>P1 vs. P150:<br>*p* <0.0001	P3 vs. P5:<br>*p* = 0.9415<br>P3 vs. P7:<br>*p* = 0.2174<br>P3 vs. P10:<br>*p* <0.0001<br>P3 vs. P15:<br>*p* <0.0001<br>P3 vs. P28:<br>*p* <0.0001<br>P3 vs. P55:<br>*p* <0.0001<br>P3 vs. P150:<br>*p* <0.0001	P5 vs. P7:<br>*p* = 0.9067<br>P5 vs. P10:<br>*p* <0.0001<br>P5 vs. P15:<br>*p* <0.0001<br>P5 vs. P28:<br>*p* <0.0001<br>P5 vs. P55:<br>*p* <0.0001<br>P5 vs. P150:<br>*p* <0.0001	P07 vs. P10:<br>*p* = 0.0037<br>P07 vs. P15:<br>*p* <0.0001<br>P07 vs. P28:<br>*p* <0.0001<br>P07 vs. P55:<br>*p* <0.0001<br>P07 vs. P150:<br>*p* <0.0001	P10 vs. P15:<br>*p* = 0.2318<br>P10 vs. P2:<br>*p* = 0.0001<br>P10 vs. P55:<br>*p* <0.0001<br>P10 vs. P150:<br>*p* <0.0001	P15 vs. P28:<br>*p* = 0.1485<br>P15 vs. P55:<br>*p* <0.0001<br>P15 vs. P150:<br>*p* <0.0001	P28 vs. P55:<br>*p* = 0.1381<br>P28 vs. P150:<br>*p* = 0.0008	P55 vs. P150:<br>*p* = 0.4086

Developmental AIS elongation in M1LV pyramidal neurons: AIS length; oneway ANOVA + *post-hoc test.* Statistical comparison of AIS length between age groups, referring to data in Supplementary Table 2. P values for post-hoc test are reported for each paired comparison. The statistical tests used for comparing these (normally distributed) samples was one-way ANOVA and Bonferroni's multiple comparisons test (A. + Bonferroni).

Supplementary Table 4						
	**P2-5 (n = 24)**	**P10-15 (n = 21)**	**P20-25 (n = 21)**	**P50-56 (n = 19)**	**P > 150 (n = 20)**	**oneway ANOVA + *post-hoc test***
**rheobase (pA)**	11.9 ± 9.5	24.5 ± 20.0	57.4 ± 46.5	75.8 ± 37.6	51.7 ± 29.0	Kruskal-Wallis test: p < 0.0001 (K-W + Dunn's multiple comparisons test:*P2-5 vs. P10-15: p = 0.8636*<br>*P2-5 vs. P20-25: p < 0.0001*<br>*P2-5 vs. P50-56: < 0.0001*<br>*P2-5 vs. P > 150: < 0.0001*<br>*P10-15 vs. P 20-25: p = 0.0560*<br>*P10-15 vs. P 50-56: p = 0.0003*<br>*P10-15 vs. P > 150: p = 0.0698*<br>*P20-25 vs. P 50-56: p > 0.9999*<br>*P20-25 vs. P > 150: p > 0.9999*<br>*P50-56 vs. P 150: p > 0.9999*
**Max gain *d*AP frequency/ dI_{input} (Hz/pA)**	$14 \times 10^{-3} \pm 7 \times 10^{-3}$	$7 \times 10^{-3} \pm 3 \times 10^{-3}$	$5 \times 10^{-3} \pm 1 \times 10^{-3}$	$7 \times 10^{-3} \pm 2 \times 10^{-3}$	$6 \times 10^{-3} \pm 2 \times 10^{-3}$	ANOVA: p < 0.0001<br>Bonferroni's multiple comparisons test:<br>*P2-5 vs. P10-15: p < 0.0627*<br>*P2-5 vs. P20-25: p = 0.0001*<br>*P2-5 vs. P50-56: p = 0.0818*<br>*P2-5 vs. P > 150: p = 0.001*<br>*P10-15 vs. P 20-25: p > 0.5937*<br>*P10-15 vs. P 50-56: p > 0.9999*<br>*P10-15 vs. P > 150: p > 0.9999*<br>*P20-25 vs. P 50-56: p = 0.7328*<br>*P20-25 vs. P > 150: p > 0.9999*<br>*P50-56 vs. P 150: p > 0.9999*
**max AP freq. (Hz)**	19 ± 14	19 ± 8	29 ± 12	30 ± 7	31 ± 8	Kruskal-Wallis test: p < 0.0001 (K-W + Dunn's multiple comparisons test:*P2-5 vs. P10-15: p > 0.9999*<br>*P2-5 vs. P20-25: p = 0.1564*<br>*P2-5 vs. P50-56: p = 0.0065*<br>*P2-5 vs. P > 150: p = 0.0023*<br>*P10-15 vs. P 20-25: p = 0.0555*<br>*P10-15 vs. P 50-56: p = 0.0019*<br>*P10-15 vs. P > 150: p = 0.0006*

						P20-25 vs. P 50-56: p > 0.9999<br>*P20-25 vs. P > 150: p > 0.9999*<br>*P50-56 vs. P 150: p > 0.9999*
**AP threshold (mV)**	-30<br>± 7	-42<br>± 7	-43<br>± 7	-42<br>± 8	-44<br>± 7	Kruskal-Wallis test: p < 0.0001 (K-W + Dunn’s multiple comparisons test:*P2-5 vs. P10-15: p = 0.0005*<br>*P2-5 vs. P20-25: p < 0.0001*<br>*P2-5 vs. P50-56: p = 0.0027*<br>*P2-5 vs. P > 150: p < 0.0001*<br>*P10-15 vs. P 20-25: p > 0.9999*<br>*P10-15 vs. P 50-56: > 0.9999*<br>*P10-15 vs. P > 150: > 0.9999*<br>*P20-25 vs. P 50-56: > 0.9999*<br>*P20-25 vs. P > 150: > 0.9999*<br>*P50-56 vs. P 150: > 0.9999*
**max dV/dT (V/s)**	95<br>± 41	158<br>± 27	254<br>± 61	219<br>± 44	218<br>± 65	Kruskal-Wallis test: p < 0.0001 (K-W + Dunn’s multiple comparisons test:P2-5 vs. P10-15: p = 0.0937<br>P2-5 vs. P20-25: p < 0.0001<br>P2-5 vs. P50-56: p < 0.0001<br>P2-5 vs. P > 150: p < 0.0001<br>P10-15 vs. P 20-25: p = 0.0002<br>P10-15 vs. P 50-56: p = 0.0184<br>P10-15 vs. P > 150: p = 0.0441<br>P20-25 vs. P 50-56: p > 0.9999<br>P20-25 vs. P > 150: p > 0.9999<br>P50-56 vs. P 150: p > 0.9999
**min dV/dT (V/s)**	-21<br>± 11	-31<br>± 7	-53<br>± 20	-52<br>± 7	-61<br>± 14	Kruskal-Wallis test: p < 0.<br>Dunn’s multiple comparisons test:*P2-5 vs. P10-15: p = 0.0529*<br>*P2-5 vs. P20-25: p < 0.0001*<br>*P2-5 vs. P50-56: p < 0.0001*<br>*P2-5 vs. P > 150: p < 0.0001*<br>*P10-15 vs. P 20-25: p < 0.0001*<br>*P10-15 vs. P 50-56: p < 0.0001*<br>*P10-15 vs. P > 150: p < 0.0001*<br>*P20-25 vs. P 50-56: p > 0.9999*<br>*P20-25 vs. P > 150: p = 0.1867*<br>*P50-56 vs. P 150: p = 0.1666*

**AP half-width (ms)**	3.5<br>± 1.6	2.3<br>± 0.4	1.7<br>± 0.5	1.7<br>± 0.2	1.4<br>± 0.3	Kruskal-Wallis test: p < 0.0001<br>Dunn's multiple comparisons test:P2-5 vs. P10-15: p > 0.9999<br>P2-5 vs. P20-25: p < 0.0001<br>P2-5 vs. P50-56: p = 0.0001<br>P2-5 vs. P > 150: p < 0.0001<br>P10-15 vs. P 20-25: p = 0.0036<br>P10-15 vs. P 50-56: p = 0.0140<br>P10-15 vs. P > 150: p < 0.0001<br>P20-25 vs. P 50-56: p > 0.9999<br>P20-25 vs. P > 150: p = 0.8198<br>P50-56 vs. P 150: p = 0.3962

Developmental changes in intrinsic membrane properties and input – output gain. Intrinsic membrane properties, maximal input-output gain, rheobase, maximal AP frequency, AP threshold, max dV/dt, min dV/dt, and AP half-width of M1LV neuron are reported as average ± standard deviation. P values for multiple comparison and post-hoc test are reported for each paired comparison. The statistical tests used for normally distributed samples were one-way ANOVA and Bonferroni's multiple comparisons test (A. + Bonferroni). Non-normally distributed samples were compared with the Kruskal-Wallis test and Dunn's multiple comparisons test (K-W + Dunn's).

Supplementary Table 5.

	**P2-5 (n = 24)**	**P10-15 (n = 21)**	**P20-25 (n = 21)**	**P50-56 (n = 19)**	**P > 150 (n = 20)**	**oneway ANOVA + *post-hoc test***
**IS (V/s)**	100 ± 40	150 ± 31	179 ± 60	180 ± 31	190 ± 47	Kruskal-Wallis test: p < 0.0001<br>Dunn's multiple comparisons test:*P2-5 vs. P10-15: p =0.1341*<br>*P2-5 vs. P20-25: p = 0.0002*<br>*P2-5 vs. P50-56: p < 0.0001*<br>*P2-5 vs. P > 150: p < 0.0001*<br>*P10-15 vs. P 20-25: p = 0.5839*<br>*P10-15 vs. P 50-56: p = 0.2295*<br>*P10-15 vs. P > 150: p = 0.0797*<br>*P20-25 vs. P 50-56: p > 0.9999*<br>*P20-25 vs. P > 150: p > 0.9999*<br>*P50-56 vs. P 150: p > 0.9999*
**SD (V/s)**	n.a.	130 ± 31	248 ± 66	250 ± 50	274 ± 52	Kruskal-Wallis test: p < 0.0001<br>Dunn's multiple comparisons test:*P10-15 vs. P 20-25: p < 0.0001*<br>*P10-15 vs. P 50-56: p < 0.0001*<br>*P10-15 vs. P > 150: p < 0.0001*<br>*P20-25 vs. P 50-56: p = 0.9998*<br>*P20-25 vs. P > 150: p > 0.4143*<br>*P50-56 vs. P 150: p > 0.4942*
**Est $I_{In\text{-}AIS}$ (pA)**	126 ± 50	273 ± 58	451 ± 148	490.5 ± 84.98	550.6 ± 136.7	Kruskal-Wallis test: p < 0.0001<br>Dunn's multiple comparisons test:*P2-5 vs. P10-15: p < 0.0001*<br>*P2-5 vs. P20-25: p < 0.0001*<br>*P2-5 vs. P50-56: p < 0.0001*<br>*P2-5 vs. P > 150: p < 0.0001*<br>*P10-15 vs. P 20-25: p = 0.0009*<br>*P10-15 vs. P 50-56: p < 0.0001*<br>*P10-15 vs. P > 150: p < 0.0001*<br>*P20-25 vs. P 50-56: p = 0.9778*<br>*P20-25 vs. P > 150: p > 0.3143*

						P50-56 vs. P 150: p > 0.6648

Developmental changes in IS and SD component. AP IS and SD component, and estimated inward current at the AIS (Est $I_{In\text{-}AIS}$) of M1LV neuron are reported as average ± standard deviation. P values for multiple comparison and post-hoc test are reported for each paired comparison. These (non-normally distributed) samples were compared with the Kruskal-Wallis test and Dunn's multiple comparisons test (K-W + Dunn's).

Supplementary Table 6.

	P2-5 (n_{mono}/n_{tri} = 21/0)	P10-15 (n_{mono}/n_{tri} = 19/0)	P20-25 (n_{mono}/n_{tri} = 20/7)	P50-56 (n_{mono}/n_{tri} = 18/6)	P > 150 (n_{mono}/n_{tri} = 16/14)	oneway ANOVA + *post-hoc test*
**$AHP_{mono/slow}$ (mV)**	19 ± 3	17 ± 3	13 ± 3	11 ± 3	14 ± 4	ANOVA: p < 0.0001<br>Bonferroni's multiple comparisons test:*P2-5 vs. P10-15: p =0.2449*<br>*P2-5 vs. P20-25: p < 0.0001*<br>*P2-5 vs. P50-56: p < 0.0001*<br>*P2-5 vs. P > 150: p = 0.0002*<br>*P10-15 vs. P 20-25: p = 0.0010*<br>*P10-15 vs. P 50-56: p < 0.0001*<br>*P10-15 vs. P > 150: p = 0.2480*<br>*P20-25 vs. P 50-56: p > 0.9999*<br>*P20-25 vs. P > 150: p > 0.9999*<br>*P50-56 vs. P 150: p = 0.0817*
**AHP_{fast} (mV)**	n.a.	n.a.	6.1 ± 2.6	13.2 ± 2.9	9.1 ± 3.7	ANOVA: p < 0.0001<br>Bonferroni's multiple comparisons test:<br>*P20-25 vs. P 50-56: p = 0.0025*<br>*P20-25 vs. P > 150: p = 0.1773*<br>*P50-56 vs. P 150: p = 0.0619*
**ADP (mV)**	n.a.	n.a.	2.2 ± 1.8	1.8 ± 1.0	3.9 ± 2.8	ANOVA: p < 0.0968<br>Bonferroni's multiple comparisons test:*P20-25 vs. P 50-56: p > 0.9999*<br>*P20-25 vs. P > 150: p = 0.3324*<br>*P50-56 vs. P 150: p = 0.1771*

Developmental changes of AHP and ADP. Average amplitude of $AHP_{mono/slow}$, AHP_{fast} and ADP of M1LV neurons are reported as average ± standard deviation. P values for multiple comparison and post-hoc test are reported for each paired comparison. Below the table headers indicating age groups, the number of neurons showing mono-phasic events (n_{mono}) and tri-phasic events (n_{tri}) are reported side by side (n_{mono}/n_{tri}). The statistical tests used for comparing these (normally distributed) samples was one-way ANOVA and Bonferroni's multiple comparisons test (A. + Bonferroni).

Supplementary Table 7.						
	**P2-5 (n = 21)**	**P10-15 (n = 19)**	**P20-25 (n = 14)**	**P50-56 (n = 15)**	**P > 150 (n = 13)**	**oneway ANOVA + *post-hoc test***
I_{In} **(nA)**	-2.0 ± 0.7	-4.6 ± 1.4	-7.5 ± 1.9	-5.4 ± 2.8	-6.6 ± 2.6	ANOVA: p < 0.0001<br>Bonferroni's multiple comparisons test:*P2-5 vs. P10-15: p =0.0005*<br>*P2-5 vs. P20-25: p < 0.0001*<br>*P2-5 vs. P50-56: p < 0.0001*<br>*P2-5 vs. P > 150: p < 0.0001*<br>*P10-15 vs. P 20-25: p = 0.0005*<br>*P10-15 vs. P 50-56: p = 0.7067*<br>*P10-15 vs. P > 150: p = 0.0303*<br>*P20-25 vs. P 50-56: p = 0.0412*<br>*P20-25 vs. P > 150: p = 0.7935*<br>*P50-56 vs. P 150: p = 0.4575*
$I_{In}V_{Half}$ **(mV)**	-34 ± 7	-47 ± 7	-51 ± 9	-48 ± 6	-46 ± 11	Kruskal-Wallis test: p < 0.0001<br>Dunn's multiple comparisons test:*P2-5 vs. P10-15: p =0.0002*<br>*P2-5 vs. P20-25: p < 0.0001*<br>*P2-5 vs. P50-56: p = 0.0001*<br>*P2-5 vs. P > 150: p = 0.0062*<br>*P10-15 vs. P 20-25: p > 0.9999*<br>*P10-15 vs. P 50-56: p > 0.9999*<br>*P10-15 vs. P > 150: p > 0.9999*<br>*P20-25 vs. P 50-56: p > 0.9999*<br>*P20-25 vs. P > 150: p > 0.9999*<br>*P50-56 vs. P 150: p > 0.9999*
I_{Out} **(nA)**	1.3 ± 0.6	3.9 ± 2.3	4.4 ± 2.0	3.6 ± 3.1	4.0 ± 1.9	Kruskal-Wallis test: p < 0.0001<br>Dunn's multiple comparisons test:*P2-5 vs. P10-15: p < 0.0001*<br>*P2-5 vs. P20-25: p < 0.0001*<br>*P2-5 vs. P50-56: p = 0.0378*<br>*P2-5 vs. P > 150: p < 0.0001*<br>*P10-15 vs. P 20-25: p > 0.9999*

						P10-15 vs. P 50-56: p > 0.9999<br>*P10-15 vs. P > 150: p > 0.9999*<br>*P20-25 vs. P 50-56: p = 0.4134*<br>*P20-25 vs. P > 150: p > 0.9999*<br>*P50-56 vs. P 150: p > 0.9999*

Developmental changes in inward and outward voltage-activated currents. Voltage sensitive inward current (I_{In}), voltage of I_{In} half-maximal activation ($I_{In}V_{Half}$), steady state outward current (I_{Out}), fast outward current ($I_{Out\ fast}$), and $I_{Out\ fast}$ decay constant (τ) of M1LV neuron are reported as average ± standard deviation. P values for multiple comparison and post-hoc test are reported for each paired comparison. The statistical tests used for normally distributed samples were one-way ANOVA and Bonferroni's multiple comparisons test (A. + Bonferroni). Non-normally distributed samples were compared with the Kruskal-Wallis test and Dunn's multiple comparisons test (K-W + Dunn's).

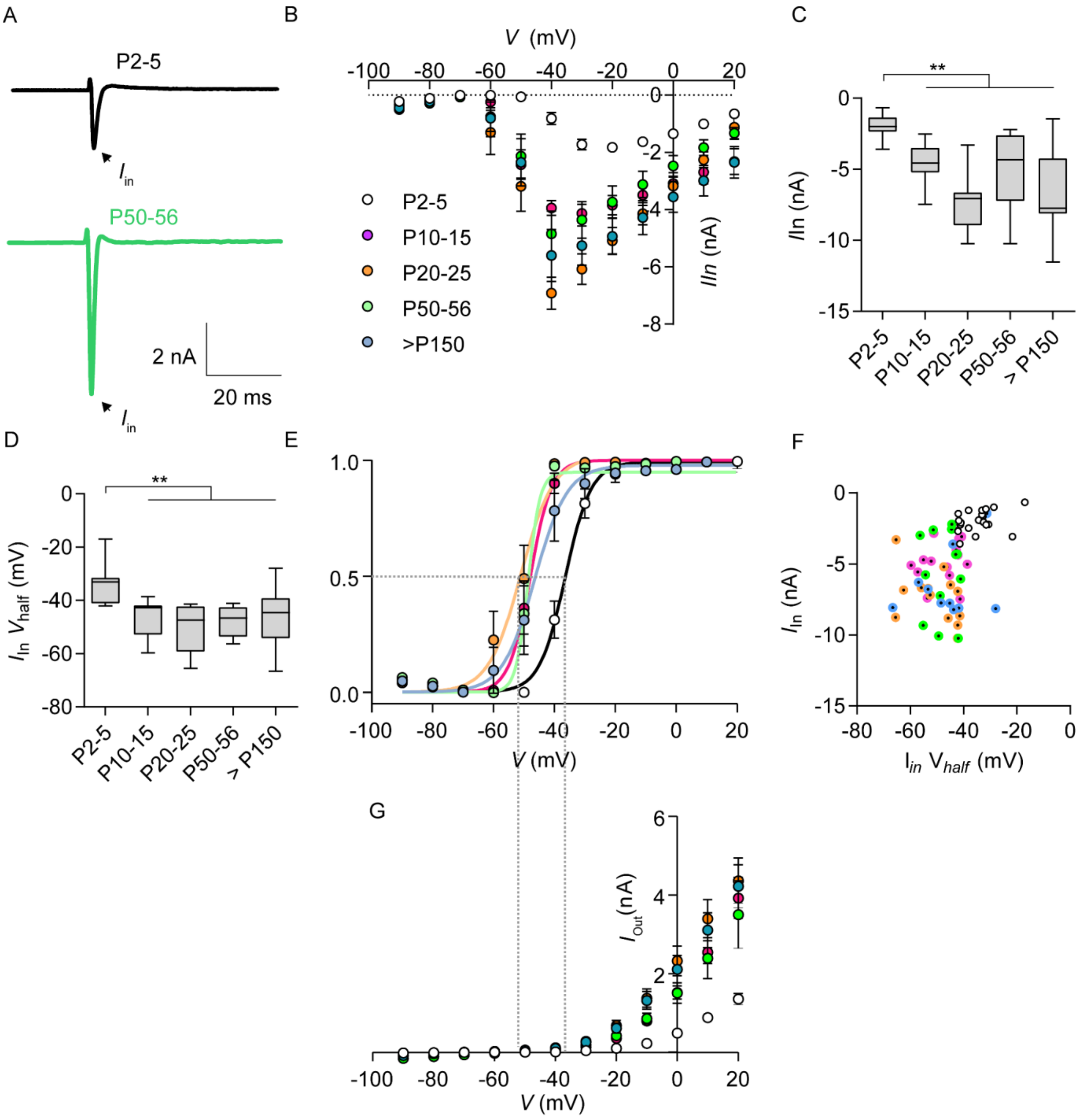

Supplementary Fig. 1 Development of voltage-activated currents. A. Typical inward current (I_{In}) of P2-5 neurons (black) and P50-56 neurons (green) upon depolarization (-70 mV to -40 mV). Peak I_{in} is highlighted by arrowheads. **B.** Current – voltage relation of peak I_{In} elicited by depolarizing steps (500 ms) of increasing voltage (from -90 mV to +20 mV, holding potential: -70 mV). **C.** Maximal amplitude of I_{In}. **D.** Inward current half-maximal activation ($I_{In}V_{half}$). **E.** Fractional activation of I_{In} for different age groups. Note the smaller voltage sensitivity at P2-5. **F.** Relation between amplitude of peak I_{In} and $I_{In}V_{half}$, samples are color coded as in panel B. **G.**

Current – voltage relation of outward currents elicited by depolarizing voltage steps (500 ms) of increasing amplitude (from -90 mV to +20 mV, holding potential: -70 mV). Note reduced amplitude and voltage dependence at P2-5. ** $p < 0.01$; P2-5: n = 21; P10-15: n = 19; P20-25: n = 14; P50-56: n = 15; P > 150: n = 13.
